# Fibromuscular dysplasia presenting as a renal infarction: a case report

**DOI:** 10.1186/1752-1947-4-199

**Published:** 2010-06-30

**Authors:** Annelies Van den Driessche, Erik Van Hul, Malika Ichiche, Gert A Verpooten, Jean-Louis Bosmans

**Affiliations:** 1 Department of Nephrology-Hypertension, Antwerp University Hospital, Edegem (Antwerpen), Belgium; 2 Department of Radiology, Antwerp University Hospital, Edegem (Antwerpen), Belgium; 3 Laboratory of Experimental Medicine and Pediatrics, University of Antwerp, Belgium

## Abstract

**Introduction:**

Fibromuscular dysplasia is a non-atherosclerotic, non-inflammatory disease that most commonly affects the renal and internal carotid arteries.

**Case presentation:**

We present the case of a 44-year-old Caucasian man who was admitted with complaints of loin pain and hypertension. A computed tomography scan of the abdomen revealed a right renal infarction with a nodular aspect of the right renal artery. Subsequent renal angiography revealed a typical 'string of beads' pattern of the right renal artery with thrombus formation. Oral anticoagulation was started and the secondary hypertension was easily controlled with anti-hypertensive drugs. At follow-up, our patient refused percutaneous transluminal renal angioplasty as a definitive treatment.

**Conclusions:**

Fibromuscular dysplasia is the most common cause of renovascular hypertension in patients under 50 years of age. Presentation with renal infarction is rare.

In fibromuscular dysplasia, angioplasty has been proven to have, at least for some indications, an advantage over anti-hypertensive drugs. Therefore, hypertension secondary to fibromuscular dysplasia is the most common cause of curable hypertension.

## Introduction

Fibromuscular dysplasia (FMD) describes a group of conditions which cause non-atheromatous arterial stenoses, most commonly of the renal and internal carotid arteries. FMD is mostly seen in young women. It typically presents with hypertension, but presentation with renal infarction has been described in a handful of cases. The clinical presentation of renal infarction includes loin pain and fever. There may be diagnostic delay before diagnosis is made. We report the case of FMD presenting with unilateral renal infarction.

## Case presentation

**Figure 1:**
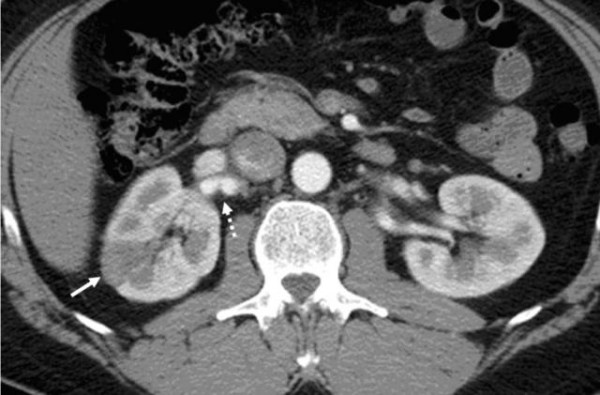
**White arrow: right renal infarction**. Dotted white arrow: nodular aspect of the right renal artery.

**Figure 2:**
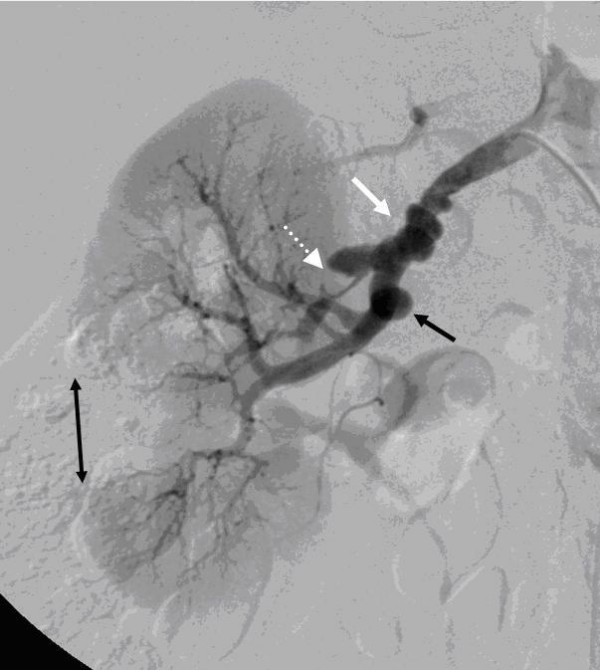
**White arrow: typical string of beads pattern of the right renal artery**. Dotted white arrow: thrombus formation in the right upper renal artery. Short black arrow: aneurysm formation. Long black arrow: renal infarction due to thrombus formation of the right middle renal artery.

A 44-year-old Caucasian (Belgian) man was admitted to the emergency room, after returning from a trip to France. Complaints of right loin pain had started two weeks earlier and were unsuccessfully treated with non-steroidal anti-inflammatory agents. His past medical history included arterial hypertension for which the patient refused medical treatment. He had already started a diet program in order to lower his blood pressure by losing weight. His blood pressure on admission was 191/106 mmHg, pulse was regular and temperature was 37.5°C. His kidney function was slightly impaired with an estimated glomerular filtration rate (GFR) of 71 mL/min/1.73 m^2 ^(abbreviated modification of diet in renal disease (MDRD) formula), a moderate leukocytosis (10,700/mm^3^) and slight elevation of C-reactive protein (CRP) (1.9 mg/dL), besides elevated plasma concentrations of lactate dehydrogenase (639 U/L). A computerized tomography (CT) scan demonstrated three wedge shaped areas of low attenuation in the right kidney, and a 'string of beads' sign of the right renal artery with suspicion of peri-arterial inflammatory changes (Figure 1). A sequential angiography with digital subtraction was performed, which confirmed the diagnosis of FMD of the right renal artery (Figure 2). In addition, an intra-luminal thrombus in the upper right renal artery was observed. Intravenous therapy with heparin was started and his blood pressure was easily controlled pharmacologically. Additional thrombophilia screening (anti-cardiolipin antibodies, lupus anticoagulant, anti-thrombin III, factor V Leiden and levels of proteins C and S) turned out to be negative. Echocardiography showed left ventricular hypertrophy with normal left ventricular function. Our patient was discharged a few days later with oral anticoagulation and the combination of two classes of anti-hypertensive drugs. During follow-up, his blood pressure remained perfectly under control. We advised the patient to undergo a new angiography in order to evaluate if the thrombus had resolved and to treat aneurysms and hemodynamically significant stenotic regions.

## Discussion

FMD is a multifactorial arteriopathy that primarily affects small and medium-sized arteries. It is most common in the renal and internal carotid arteries, but has been described in almost every arterial bed in the body.

The first description of FMD has been attributed to Leadbetter and Burkland in 1938 [1]. The term fibromuscular dysplasia was introduced in 1958 in McCornack and associates' description of three patients with hypertension and renal artery stenosis [2].

FMD is most often diagnosed in the renal arteries, accounting for 60 to 75% of cases. Involvement of extra-cranial cerebrovascular arteries accounts for 25 to 30% of cases, and miscellaneous other arteries (mesenteric or brachial arteries) for up to 30%. Approximately 25% of patients have multiple arteries involved. The exact incidence of asymptomatic FMD throughout the arterial circulation is unknown. Renal artery involvement has been identified through angiographic evaluation of asymptomatic, non-hypertensive potential renal donors by two study groups [3,4]. The reported prevalence was 4.4 to 6.6%. Among adults, FMD is more common among women, with a prevalence two to ten times higher compared to men. There does not appear to be a female predominance in children [5]. Most cases of FMD are diagnosed in patients younger than 50 years, with the exception of FMD of the cerebral circulation. FMD of the renal arteries is bilateral in 35 to 50%, and among those with bilateral disease, nearly half have extra-renal involvement [3,5].

The cause of FMD is unknown and is probably multifactorial. The most commonly held hypothesis includes hormonal, ischemic, mechanical and genetic factors.

The pathological classification is based on the predominantly involved arterial wall layer. The lesions generally are classified into four types. Macro-aneurysms and dissections are complications of FMD, and do not represent distinct histopathological categories.

Medial fibroplasia is characterized by the classic angiographical 'string of beads' appearance, and represents the most common dysplastic lesion. It is typically located in the middle-to-distal portion of the artery. Intimal fibroplasia occurs in less than 10% of patients with arterial fibrodysplasia. Angiographically, it may appear as a focal, concentric stenosis and is often bilateral. Perimedial dysplasia accounts for 10% of the lesion in renal arteries. On angiography, multiple high-grade stenoses of the main renal artery without aneurysmal dilatation are seen.

Adventitial hyperplasia is the rarest type of fibrodysplastic lesion. There is limited angiographic information. Sharply localized, tubular areas of stenosis have been observed.

Renal infarction in the general population commonly results from thromboembolism due to structural or arrythmical cardiac disease, but it is also associated with coagulation disorders, vasculitis, trauma and conditions of blood vessel dysfunction, such as Marfan syndrome, Ehlers-Danlos syndrome and, as presented in this case, FMD [6-8]. Progression to renal infarction in FMD is rare. Most patients who develop renal infarction have perimedial dysplasia [9]. In perimedial dysplasia, large parts of the media are replaced by collagen, with irregular thickening of the media which may lead to total occlusion. Also intimal dysplasia seems to have a worse prognosis. It is caused by circumferential or eccentric deposition of collagen in the intima and usually affects multiple renal artery braches. So, it is more likely to develop dissections or dangerous ischemic nephropathy [10]. But also in medial fibrodysplasia renal infarction has been reported. In this type of FMD, there are alternating fibromuscular webs and aneurysmal dilatation. The aneurysms seen in FMD are all true aneurysms. Renal infarction can result from embolization from the aneurysm sac. In areas of alternating stenosis and dilatation, shear-induced platelet activation precipitates thrombus formation [11].

In most cases, individuals with FMD have been asymptomatic for many years and the diagnosis is made incidentally during the investigation of another problem. Disease manifestations may result from ischemia related to stenosis, spontaneous dissection of arteries, rupture of aneurysms or embolization of intra-vascular thrombi from aneurysmal segments. The primary clinical manifestation of renal FMD is hypertension. Often, hypertension tends to be refractory to simple drug therapy. As in atherosclerotic renovascular occlusive disease, use of an angiotensin-converting enzyme (ACE)-inhibitor can often worsen renal function in bilateral disease. Loss of renal function occurs in up to 63% of patients with renal artery FMD, but overt renal failure is rare in these patients. FMD presenting as a renal infarction is quite exceptional. Flank pain occurs in the majority of these patients. Fever, vomiting, and oliguria constitute other symptoms. Hematuria arises in roughly 50% of cases. Lactate dehydrogenase is elevated in most cases.

The gold standard for evaluating renal artery FMD remains digital subtraction angiography.

The main impetus for the treatment of FMD is control of hypertension. The treatment for most patients can be primarily managed medically [12]. The principles of drug therapy are based on the pathophysiology of renal artery stenosis and the Renin-Angiotensin-Aldosterone-System (RAAS). In patients with unilateral stenosis, renin-angiotensin mediated vasoconstriction is the primary mechanism of hypertension. The normal contra-lateral kidney is able to produce a natriuresis, thus avoiding aldosterone-mediated volume retention. In case of bilateral stenoses, the natriuresis cannot occur, leading to sodium and volume retention, which is the main mechanism of hypertension in these patients. In conclusion, ACE-inhibitors should be first-line agents in the former case, as diuretics should be in the latter form.

Percutaneous transluminal renal angioplasty (PTRA) should be considered in well-defined groups of patients: those with a recent onset of hypertension (in particular patients younger than 50 who are less likely to have underlying atherosclerotic disease) in whom the goal is to cure the hypertension; those in whom blood pressure control had proved to be difficult despite the use of a combination of anti-hypertensive drugs; those with an intolerance to anti-hypertensive medications and those who have lost renal volume because of ischemic nephropathy. In patients with relatively well-controlled hypertension, or with no loss of renal parenchymal mass, the risk of the procedure may outweigh the benefits. Angioplasty is generally sufficient and stenting is only performed when there is a suboptimal balloon result or flow-limiting dissection. The reported success rates for PTRA range from 83 to 100% [12-17]. Overall cure and improvement/benefit rates for hypertension vary from 22 to 94% and depend on the definition that is given to 'cured' and 'improved' [17-22]. Comparison between studies is therefore not easy and often inaccurate. The rate of restenosis, following PTRA ranges from 6.7 to 23% [12,14,15,17-19]. Complications of angioplasty include renal artery dissection and perforation, contrast nephropathy, and hematoma or pseudo-aneurysm formation at the access site. Some authors mention macro-aneurysms, branch vessel disease and complex dissections as contra-indications for PTRA [13]. Others claim that PTRA is equally effective in the main renal artery and in branch-artery stenoses [17]. A recent retrospective study showed that long duration of hypertension, high age and FMD involvement of branch arteries negatively affect efficacy of treatment [16]. On the other hand, predictors of long-term clinical benefit seem to be: duration of hypertension < 8 years, serum creatinine <1.5 mg/dL and functional status of the contra-lateral kidney.

## Conclusions

In this case report, we presented a case of renal infarction complicating FMD. This rare combination has only been described in a handful of cases [21,22]. The typical features of acute renal infarction include persistent abdominal, flank or lower back pain. Early recognition is primordial in this patient group (predominantly less than 50 years of age). In our patient, blood pressure was easily controlled by two classes of oral anti-hypertensive agents. During follow-up, blood pressure remained perfectly controlled. However, a redo-angiography has to be performed, as it is important to control if thrombus has resolved. Moreover, it is necessary to assess hemodynamic significance of the renal artery stenosis and if so, to perform a dilatation. In particular, *de novo *embolization and recurrent renal infarction has to be prevented. Unfortunately, our patient refused redo-angiography.

## Consent

Written informed consent was obtained from the patient for publication of this case report and any accompanying images. A copy of the written consent is available for review by the Editor-in-Chief of this journal.

## Conflict of interest statement

The authors declare that they have no competing interests.

## Authors' contributions

AVDD wrote the article. JLB was a major contributor in writing the manuscript. EVH made the radiological diagnosis. AVDD, MI, GV and JLB all contributed to clinical diagnosis and work-up. All authors read and approved the final manuscript.
